# Epe1 contributes to activation of AMPK by promoting phosphorylation of AMPK alpha subunit, Ssp2

**DOI:** 10.1038/s41598-017-03442-0

**Published:** 2017-06-09

**Authors:** Yongyi Chen, Xiaoyue Hu, Chao Guo, Yao Yu, Hong Lu

**Affiliations:** 1 State Key Laboratory of Genetic Engineering, School of Life Sciences, Fudan University, Shanghai, 200438 China; 2 Shanghai Engineering Research Center of Industrial Microorganisms, Shanghai, 200438 China; 3 Shanghai Collaborative Innovation Center for Biomanufacturing Technology, Shanghai, 200237 China

## Abstract

AMP-activated protein kinase (AMPK) is a pivotal cellular energy sensor. It is activated by stresses that cause depletion of energy and initiates adaptive responses by regulating metabolism balance. AMPK forms αβγ heterotrimer. In fission yeast, activation of AMPK mainly depends on the phosphorylation of AMPKα subunit Ssp2 at Thr^189^ by upstream kinase Ssp1. However, not much is known about the regulation of this process. In this study, we identified Epe1 as a novel positive regulator of AMPK. Epe1, a jmjC-domain-containing protein, is best-known as a negative regulator of heterochromatin spreading. Although the novel role of Epe1 in regulation of AMPK relies on predicted iron- and 2-oxyglutarate-binding residues inside jmjC domain, it seems to be irrelevant to inhibition of heterochromatin spreading. Epe1 is associated with Ssp2 directly and promotes phosphorylation of Ssp2 upon various environmental stresses, including low-glucose, high-sodium, high-pH and oxidative conditions. Similar to Epe1, Jmj1 and Msc1 also contribute to phosphorylation of Ssp2. Deletion of *epe1* ^+^ impairs downstream events following phosphorylation of Ssp2, including nuclear translocation of Ssp2, sexual differentiation and inhibition of fatty acid synthesis. Our study reveals a novel way in which a jmjC-domain-containing protein regulates adaptive response by directly binding to a principal sensor.

## Introduction

AMP-activated protein kinase (AMPK) is a principal sensor of cellular energy status and initiates adaptive responses upon energy limitation. AMPK is expressed in essentially all eukaryotic cells as heterotrimeric complexes consisting of catalytic α subunits and regulatory β and γ subunits. In mammals, AMPK is activated by stresses that cause depletion of cellular ATP and hence elevation of AMP and ADP levels, including starvation for glucose or oxygen, muscle contraction, and metabolic poisons^1^. AMP and ADP bind to the γ subunit and promote phosphorylation of Thr172 in the activation loop of α subunit by kinases LKB1 or CaMKK. Phosphorylation causes at least a 100-fold increase in activity of AMPK, whereas allosteric activation by AMP is about 10-fold^2^. Once activated, AMPK turns on catabolic pathways and turns off ATP-consuming pathways by phosphorylating and regulating key enzymes in all branches of metabolism, including fatty acid and sterol synthesis, sugar metabolism, protein synthesis, and DNA replication^3^.

Owing to its genetically tractable property, fission yeast emerges as an important model for AMPK study. Fission yeast AMPK is composed of catalytic α subunit Ssp2, β subunit Amk2 and γ subunit Cbs2^4^. Upon glucose and nitrogen starvation, Ssp2 is phosphorylated at Thr^189^ (equivalent to Thr^172^ in mammals) by a CaMKK homolog, Ssp1. Phosphorylated AMPK accumulates in the nucleus and allows nuclear localization of the Ste11 transcription factor to promote sexual differentiation. Consistently, *ssp2*Δ and *ssp1*Δ mutants are defective in mating and sporulation^5^. *cbs2*Δ and *ssp1* ^ts^ mutants display decreased growth in low glucose^6, 7^. *ssp1*Δ cells are hypersensitive to low pH and KCl stress at high temperature^8^. This suggests a role of AMPK in the response to environmental stresses is conserved in fission yeast. Although ATP and AMP directly bind to γ subunit Cbs2, it is unclear whether this nucleotide binding can increase activity of AMPK as in mammals^4, 9^. Little is known about the regulation of AMPK activity in fission yeast.

In this study, we revealed that Epe1 is a novel positive regulator of AMPK. Epe1 is a jmjC domain-containing protein. Epe1 prevents heterochromatin from spreading beyond its natural borders and limits ectopic heterochromatin formation. The novel role of Epe1 in regulation of AMPK relies on its predicted catalytic residues inside the jmjC domain, but seems to be irrelevant to inhibition of heterochromatin spreading. Epe1 directly associates with the AMPK catalytic subunit Ssp2 and promotes its phosphorylation upon various environmental stresses. Deletion of *epe1* ^+^ abolishes nuclear accumulation of Ssp2 and downstream events, including sexual differentiation and inhibition of fatting acid synthesis. Our results highlight the role of a jmjC domain-containing protein in adaptive responses upon stresses.

## Results

### Epe1 is involved in adaptive responses to environmental stresses

**Figure 1:**
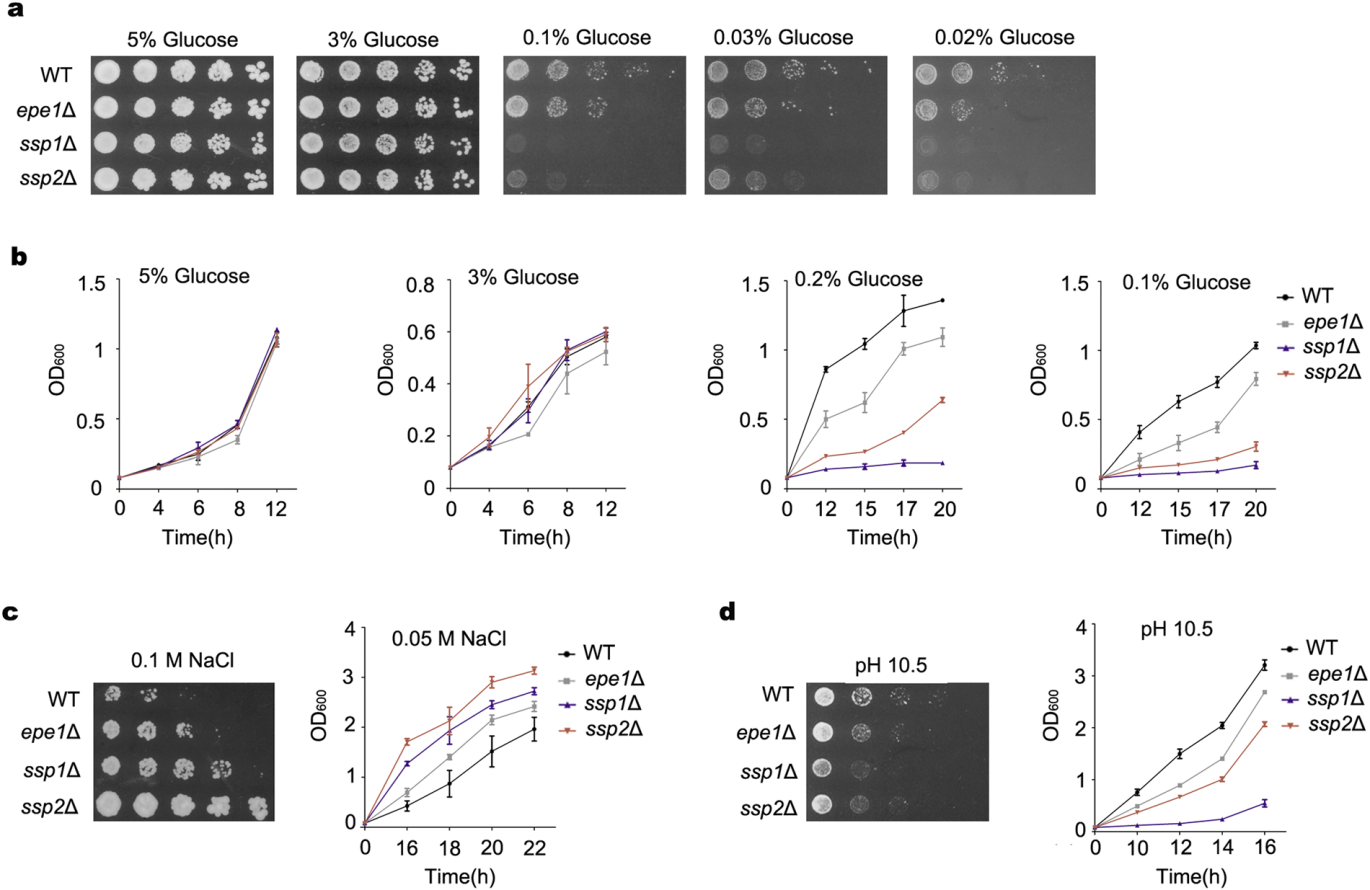
Deletion of *epe1* ^+^ impairs adaptive responses to environmental stresses. (**a**) Growth of WT, *epe1*Δ, *ssp1*Δ and *ssp2*Δ cells on low-glucose solid medium in serial dilution assays. (**b**) Growth of WT cells and mutants in low-glucose liquid medium. Values in (**b**) and below represent mean ± s.d. from three parallel cultures. (**c**) Growth of WT cells and mutants on medium containing 0.1 M NaCl or in liquid medium containing 0.05 M NaCl. (**d**) Growth of WT cells and mutants at pH 10.5.

In a genome wide screen, *epe1*Δ mutant displayed decreased growth in low-glucose medium. Epe1 is best-known as a negative regulator of heterochromatin spreading^10, 11^. However, its role in response to limited glucose was unclear. Thus, the growth of an *epe1*Δ mutant in medium containing different concentrations of glucose was investigated. As shown in Fig. 1a, Epe1 displayed normal growth on high-glucose medium (5% or 3%). The growth of *epe1*Δ mutant was reduced slightly on medium containing 0.1% glucose, and the growth defect became more apparent in even lower concentrations of glucose (0.03% and 0.02%). More severe growth defects were observed in mutants that abolish AMPK activity, including deletion of *ssp2* ^+^ (encoding the catalytic α subunit), and deletion of *ssp1* ^+^ (encoding a CaMKK homolog that phosphorylates Ssp2 at Thr^189^). This is consistent with an essential role of AMPK in the adaptation to glucose limitation^7, 12^. Intriguingly, the growth of the *ssp1*Δ mutant was poorer than that of the *ssp2*Δ mutant, suggesting that Ssp1 regulates factor(s) other than AMPK in the response to limited glucose. Impaired growth of *ssp1*Δ, *ssp2*Δ and *epe1*Δ mutants in low-glucose conditions was further confirmed by monitoring growth curves in liquid medium. The results were consistent with those on solid medium (Fig. 1b).

Besides low-glucose condition, budding yeast AMPK is required for tolerance to a broad range of stresses, including salt, alkaline, and oxidative stress^12^. The growth of *epe1*Δ, *ssp2*Δ and *ssp1*Δ cells in the presence of high sodium was investigated. Unexpectedly, *ssp2*Δ and *ssp1*Δ cells displayed better growth than WT cells, on solid medium containing 0.1 M NaCl, or in liquid medium containing 0.05 M NaCl (Fig. 1c). This result contradicts the defective growth reported for an *ssp1*Δ mutant in 1 M KCl^8^. It suggests Ssp1, along with AMPK, play different roles in responding to high-sodium and high-potassium stresses. It is possible that AMPK induces mitotic delay upon high-sodium stress. Disruption of AMPK activity by deletion of *ssp2* ^+^ or *ssp1* ^+^ might bypass the mitotic delay and result in improved growth. Similarly, *epe1*Δ cells grew better in medium containing high concentrations of NaCl, but improvement of growth of *epe1*Δ cells was weaker than that observed in *ssp2*Δ and *ssp1*Δ mutants.

The growth of *epe1*Δ, *ssp2*Δ and *ssp1*Δ cells at high pH was also examined. *ssp2*Δ and *ssp1*Δ mutants showed a marked reduction of growth at pH 10.5. The *epe1*Δ mutant also displayed defective growth, but to a lesser extent (Fig. 1d). Since the *epe1*Δ mutant shares similar phenotypes with *ssp1*Δ or *ssp2*Δ mutants in responding to various environmental stresses, it suggests Epe1 is involved in the AMPK pathway. Epe1 is more likely a regulatory factor, owing to its relative weaker phenotype.

### Epe1 promotes phosphorylation of Ssp2 upon various stresses

**Figure 2:**
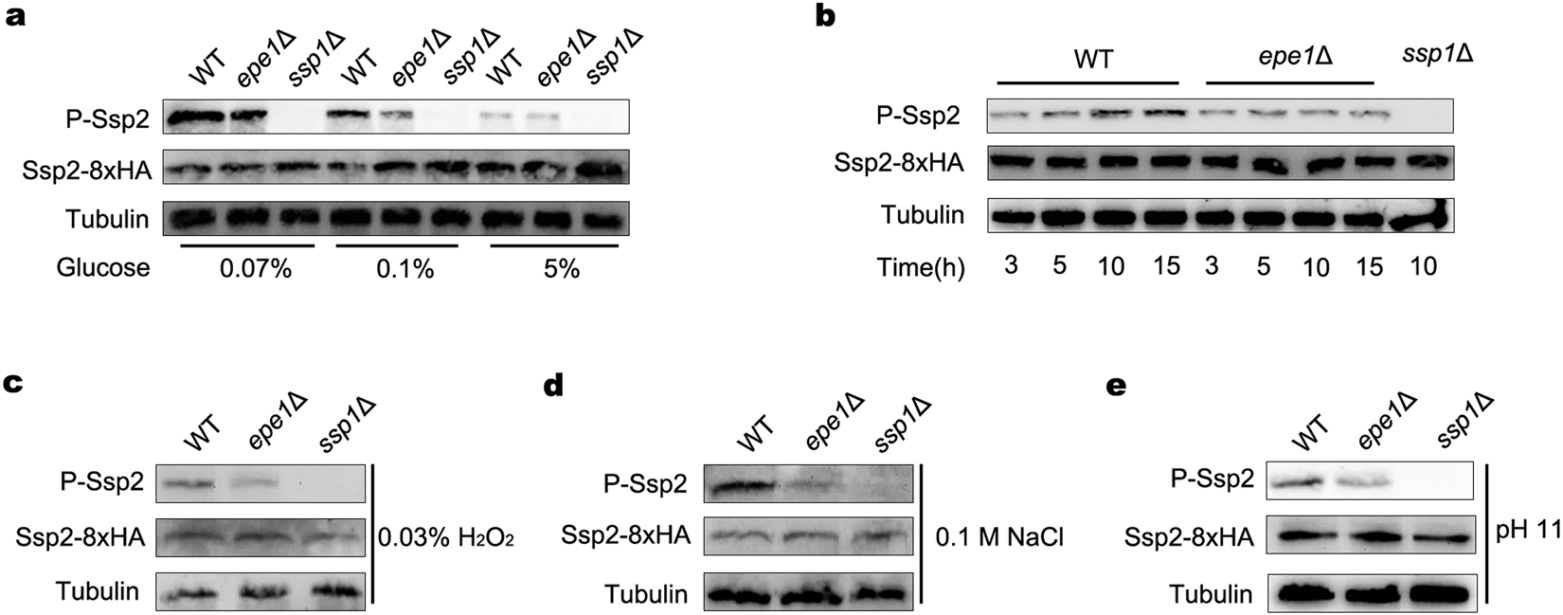
Deletion of *epe1* ^+^ abolished induction of Ssp2 phosphorylation upon environmental stresses. (**a**,**c**–**e**) Western blot to examine the amount of phosphorylated Ssp2 (P-Ssp2) in WT, *epe1*Δ, *ssp1*Δ cells growing in YES containing different concentrations of glucose (**a**), 0.03% H_2_O_2_ (**c**), 0.1 M NaCl (**d**) or at pH 11 (**e**). Tubulin was detected as a loading control. Cropped blots are shown for clarity. Full-size blots are presented in Supplementary Figures S1, S3–S5. (**b**) Western blot to examine amount of P-Ssp2 in WT, *epe1*Δ, *ssp1*Δ cells after growing in YES for 3, 5, 10 or 15 h. Cropped blots are shown for clarity. Full-size blots are shown in Supplementary Figure S2.

In fission yeast, activation of AMPK depends on the phosphorylation of Ssp2 Thr^189^ by upstream kinase Ssp1^5^. To verify a potential role of Epe1 in AMPK activation, the phosphorylation of Ssp2 upon glucose limitation was investigated by using a Phospho-AMPKα (Thr^172^) monoclonal antibody. As shown in Fig. 2a, levels of Ssp2 phosphorylation in high-glucose condition (5%) was kept low in both WT and *epe1*Δ cells. In the presence of 0.1% glucose, phosphorylation in WT was induced, and the induction was milder in *epe1*Δ mutant. In the presence of 0.07% glucose, phosphorylation of Ssp2 was induced markedly in WT cells, but was induced slightly in *epe1*Δ cell, which resulted in a substantial difference between WT and *epe1*Δ cells. As a control, phosphorylation of Ssp2 was totally abolished in an *ssp1*Δ mutant, indicating the antibody specifically recognizes phosphorylated Thr^189^ in fission yeast. The result suggests Epe1 is required for complete induction of Ssp2 Thr^189^ phosphorylation in low-glucose condition.

The effect of Epe1 on the phosphorylation of Ssp2 upon other stresses was also investigated. The level of Ssp2 Thr^189^ in oxidative (0.03% H_2_O_2_), high-sodium (1 M NaCl), or high-pH (pH 11) stresses was dramatically decreased in an *epe1*Δ mutant compared to that in WT cells, suggesting the induction of phosphorylation was impaired by the deletion of *epe1* ^+^ (Fig. 2c–e). In standard YES medium, phosphorylation of Ssp2 in WT cells was induced rapidly over time upon the depletion of nutrients, including glucose and nitrogen. In contrast, no apparent induction was observed in *epe1*Δ mutant (Fig. 2b). Thus, the results indicate Epe1 promotes the induction of Ssp2 Thr^189^ phosphorylation upon various environmental stresses.

### Epe1 is associated with Ssp2 *in vivo*

**Figure 3:**
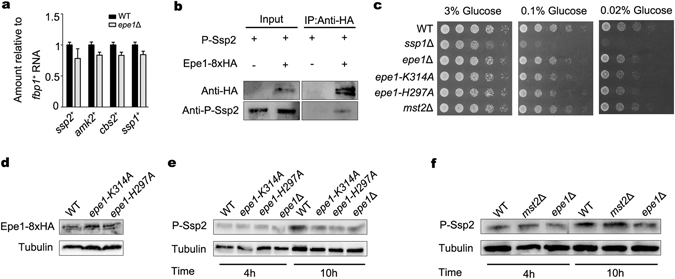
Epe1 is associated with Ssp2 *in vivo* and novel role of Epe1 requires predicted iron- and 2-oxyglutarate-binding residues inside jmjC domain. (**a**) RT-PCR analysis of mRNA levels of *ssp2* ^+^, *amk2* ^+^, *cbs2* ^+^ and *ssp1* ^+^ relative to a control *fbp1* ^+^. The relative level in WT cells was arbitrarily designated as 1. Each column represents the mean ± s.d. from three biological repeats. (**b**) Co-immunoprecipitation assay to analyze the association between Epe1 and Ssp2. Epe1-8xHA immunoprecipitation was followed by the Western blot of phosphorylated Ssp2 (P-Ssp2). Cropped blots are shown for clarity. Full-size blots are shown in Supplementary Figure S6. (**c**) Growth of WT, *ssp1*Δ, *epe1*Δ, *epe1*-*K314A*, *epe1*-*H297A* and *mst2*Δ cells on low-glucose medium in serial dilution assays. (**d**) Western blot to examine the protein levels of Epe1, Epe1-K314A and Epe1-H297A. Full-size blots are shown in Supplementary Figure S7. (**e**) Western blot to examine amount of P-Ssp2 in WT, *epe1*Δ, *epe1*-*K314A* and *epe1*-*H297A* cells after growing in YES for 4 or 10 h. Tubulin was detected as a loading control. Full-size blots are shown in Supplementary Figure S8. (**f**) Western blot to examine amount of P-Ssp2 in WT, *mst2*Δ and *epe1*Δ cells after growing in YES for 4 or 10 h. Full-size blots are shown in Supplementary Figure S9.

The mechanism underlying Epe1-promoted phosphorylation of Ssp2 was investigated. Deletion of *epe1* ^+^ causes formation of ectopic heterochromatin and results in silencing of genes in euchromatin^13^. To examine whether the deletion of *epe1* ^+^ represses the transcription of AMPK subunits and upstream kinase, an RT-PCR assay was performed. As shown in Fig. 3a, there was no significant difference between the mRNA level of *ssp2* ^+^, *cbs2* ^+^, *amk2* ^+^ or *ssp1* ^+^ in WT and *epe1*Δ cells. Accordingly, the protein level of Ssp2 in an *epe1*Δ mutant was the same as that in WT cells in regular culture or in environmental stress (Fig. 2). It suggests Epe1 does not regulate phosphorylation of Ssp2 by affecting the transcription of components of AMPK. In a previous study, fragments of Ssp2 were found by mass spectrometry in affinity purification of Epe1-FLAG^14^. Consistently, phosphorylated Ssp2 co-purified with Epe1-8xHA in a co-immunoprecipitation assay (Fig. 3b). The result suggests Epe1 promotes Ssp2 phosphorylation through a direct interaction.

### Role of Epe1 in regulation of AMPK requires its predicted iron- and 2-oxyglutarate-binding residues in jmjC domain

Epe1 blocks the expansion of heterochromatin into neighboring euchromatin and prevents formation of ectopic heterochromatin^10, 11^. Although no demethylase activity has been detected for Epe1, the predicted iron-binding residue (H297) and 2-oxyglutarate-binding residue (K314) in the jmjC domain are essential for function of Epe1 in preventing heterochromatin spreading^11^. To investigate the contribution of both residues to the role of Epe1 in AMPK regulation, H297A and K314A point mutants were constructed. Epe1-H297A and Epe1-K314A were expressed from native promoters and their levels are similar to that of wild-type Epe1 (Fig. 3d). Intriguingly, the phenotypes of Epe1-H297A and Epe1-K314A were indistinguishable from *epe1*Δ mutant. Epe1-H297A and Epe1-K314A displayed a growth defect in low-glucose condition (Fig. 3c). Accordingly, the induction of Ssp2 phosphorylation upon the depletion of nutrients was impaired in either mutant (Fig. 3e). Thus, the predicted iron- and 2-oxyglutarate-binding residues are essential for the role of Epe1 in the AMPK pathway. The role of Epe1 in preventing heterochromatin spreading is redundant with Mst2, a H3K14 acetyltransferase^13^. Deletion of *mst2* ^+^ did not cause a growth defect on low-glucose plate or delayed induction of Ssp2 phosphorylation (Fig. 3c,f). Thus, the role of Epe1 in the AMPK pathway seems unrelated to its role in limiting heterochromatin spreading.

### Jmj1 and Msc1 contribute to phosphorylation of Ssp2 and activation of AMPK pathway

**Figure 4:**
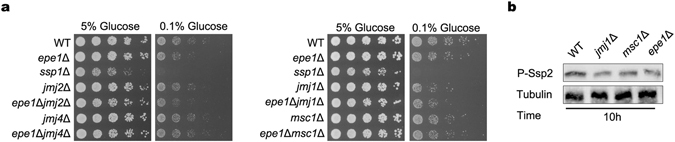
Jmj1 and Msc1 contribute to phosphorylation of Ssp2 and activation of AMPK pathway. (**a**) Growth of WT, *jmj2*Δ, *jmj2*Δ*epe1*Δ, *jmj4*Δ, *jmj4*Δ*epe1*Δ, *jmj1*Δ, *jmj1*Δ*epe1*Δ, *msc1*Δ and *msc1*Δ*epe1*Δ cells on low-glucose solid medium in serial dilution assays. (**b**) Western blot to examine amount of P-Ssp2 in WT, *jmj1*Δ, *msc1*Δ and *epe1*Δ cells after growing in YES for 10 h. Cropped blots are shown for clarity. Full-size blots are shown in Supplementary Figure S10.

Besides Epe1, Jmj1, Jmj2, Jmj3, Jmj4, Lid2 and Msc1 also contain a JmjC domain. Because *jmj3*Δ and lid2Δ mutants were both inviable, neither mutant was investigated for growth in this study. As shown in Fig. 4a, *jmj2*Δ and *jmj4*Δ mutants both grew as well as WT cells in low concentration of glucose. Similar to *epe1*Δ mutant, the growth of *jmj1*Δ and *msc1*Δ mutants was reduced slightly in low-glucose condition. The growth defect of *epe1*Δ*jmj1*Δ or *epe1*Δ*msc1*Δ mutant was about the same as *epe1*Δ mutant. Accordinly, deletion of *jmj1* ^+^ and *msc1* ^+^ reduced phosphorylation of Ssp2 upon depletion of nutrients, to a similar extent as deletion of *epe1* ^+^ (Fig. 4b). The results suggest Jmj1 and Msc1 also contribute to the activation of AMPK, and they probably function in the same pathway as Epe1.

### Epe1 is required for sexual differentiation and inhibition of fatty acid synthesis upon depletion of nutrients

**Figure 5:**
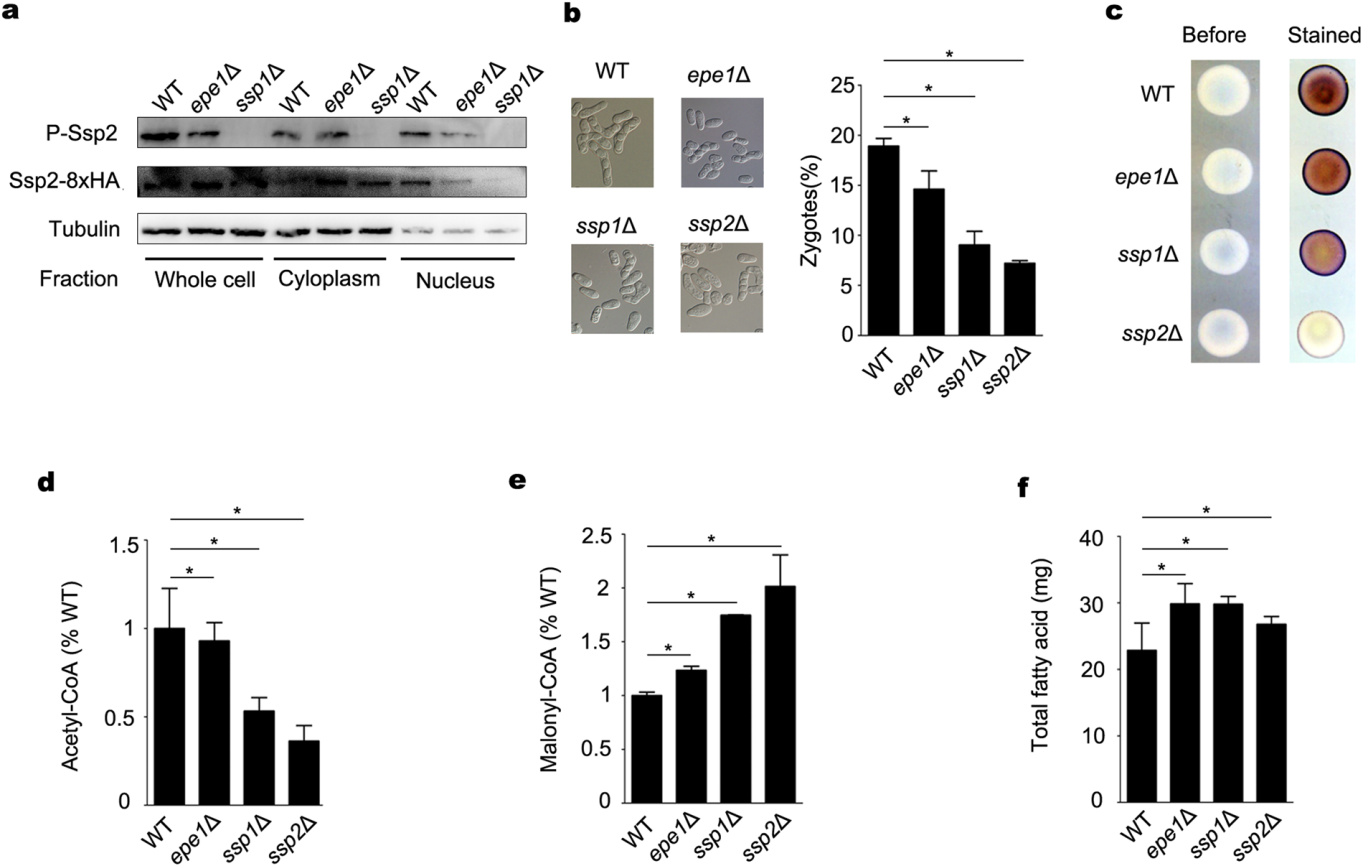
Deletion of *epe1* ^+^ impairs nuclear translocation of Ssp2, sexual differentiation and inhibition of fatty acid synthesis upon depletion of nutrients. (**a**) Western blot to examine the amount of phosphorylated Ssp2 (P-Ssp2) and Ssp2-8xHA in the cytoplasmic and nuclear fractions from WT, *epe1*Δ, *ssp1*Δ or *ssp2*Δ cells after growing in YES for 10 h. Tubulin was detected as a loading control. Cropped blots are shown for clarity. Full-size blots are shown in Supplementary Figure S11. (**b**) Percentage of zygotes in WT, *epe1*Δ, *ssp1*Δ or *ssp2*Δ cells after growing on SPAS medium for 24 h. Representative images are shown on the left and percentage is shown on the right. More than 200 cells were counted for each sample. Each column in (**b**) and below represents means ± SD from 3 biological repeats. Asterisk shown in Fig. 5b and below indicates *p* < 0.05. (**c**) Iodine staining of cells from (**b**). (**d**,**e**) Amount of acetyl-CoA (**d**) and malonyl-CoA(**e**) in WT, *epe1*Δ, *ssp1*Δ or *ssp2*Δ cells. Amount in WT cells was arbitrarily designated as 1. (**f**) Amount of total fatty acid in WT, *epe1*Δ, *ssp1*Δ or *ssp2*Δ cells.

Next, we examined the effect of Epe1 on the downstream events following phosphorylation of Ssp2. Under nutritional stress, phosphorylated AMPK is translocated into the nucleus and promotes sexual differentiation^5^. Whole cell extracts, cytoplasmic and nuclear fractions were prepared from cells after 10-hour growth. Amount of Ssp2-8xHA in whole cell lysate were about the same in WT, *epe1*Δ and *ssp1*Δ cells. In WT cells, a strong signal of Ssp2-8HA was detected in the nuclear fraction, compared to a weak signal in the cytoplasmic fraction. Nuclear accumulation of phosphorylated Ssp2 (P-Ssp2) was also observed in WT cells (Fig. 5a). In *epe1*Δ cells, the signals of Ssp2-8xHA and P-Ssp2 in the cytoplasmic fraction were much stronger than those in the nuclear fraction, indicating the nuclear translocation of Ssp2 is impaired by the deletion of *epe1* ^+^ (Fig. 5a). As a control, nuclear accumulation of Ssp2-8xHA was totally abolished in *ssp1*Δ cell, indicating the phosphorylation is essential for nuclear translocation of Ssp2 (Fig. 5a). To evaluate the role of Epe1 in sexual differentiation, mating efficiency was measured in the cells after growing on SPAS medium for 24 hours. As expected, the percentage of zygotes in *ssp2*Δ (7%) or *ssp1*Δ cells (10%) was significantly lower than that in WT cells (18%) (Fig. 5b). Deletion of *epe1* ^+^ caused a relative mild, but still significant decrease in the percentage of zygotes (15%). Consistently, *epe1*Δ cells exhibited a reduction of iodine staining comparing to WT cells, suggesting Epe1 is required for the sexual differentiation upon depletion of nutrients (Fig. 5c).

To maintain energy balance, AMPK conserves ATP by switching off anabolic pathways, including fatty acid synthesis^15^. In mammals and budding yeast, AMPK inhibits fatty acid synthesis by inactivating acetyl-coA carboxylase (Acc1), which catalyzes the irreversible carboxylation of acetyl-CoA to produce malonyl-CoA^16, 17^. To examine whether fission yeast AMPK functions in a conserved way, levels of acetyl-CoA and malonyl-CoA in WT, *epe1*Δ, *ssp1*Δ and *ssp2*Δ cells were measured after growing in the YES for 10 hours. The level of acetyl-CoA decreased and that of malonyl-CoA increased significantly in *epe1*Δ, *ssp1*Δ and *ssp2*Δ cells, compared to that in WT cells (Fig. 5d,e). The results suggest inhibition of acetyl-CoA carboxylase is impaired in the mutants. Malonyl-CoA serves as a precursor for fatty acid synthesis^18^. Accordingly, the amount of total fatty acid was significantly increased in *epe1*Δ, *ssp1*Δ and *ssp2*Δ cells (Fig. 5f). The results suggest fission yeast AMPK shares functional homology with its counterparts in mammals and budding yeast in inhibiting fatty acid synthesis. Fission yeast AMPK inactivates acetyl-coA carboxylase under nutritional stress, and this process might be positively regulated by Epe1.

## Discussion

JmjC-domain-containing proteins are implicated in chromatin modification and transcriptional regulation, where in most case their roles are dependent on the demethylase activity of the JmjC domain. Ndy1, a jmjC-domain-containing demethylase, is required for the transcription of antioxidant enzymes. Overexpression of Ndy1 relieves oxidative stress and inhibits the phosphorylation of AMPK^19^. In this study, Epe1 was identified as a novel positive regulator of AMPK. Epe1 does not promote activity of AMPK through transcriptional regulation. Instead, Epe1 is associated with Ssp2 directly and promotes the phosphorylation of Ssp2 at Thr^189^ by Ssp1. Our results reveal a novel way in which a JmjC domain-containing protein regulates adaptive responses by directly binding with a principal sensor. Whether a similar mechansim was applied in Jmj1 and Msc1-mediated regulation of AMPK is worth futher investigation.

Although no demethylase activity has been reported for the Epe1, Epe1-promoted Ssp2 phosphorylation requires the predicted iron- and 2-oxyglutarate binding residues in the jmjC domain of Epe1. Thus, it is tempting to speculate that Epe1 demethylates AMPK subunits through its jmjC domain and thus demethylation facilitates the phosphorylation of Ssp2. Activity of AMPK is regulated by various modifications. Besides phosphorylation inside the activation loop, AMPKα subunits are regulated by ubiquitination, sumoylation and oxidation/reduction. AMPKβ is subjected to acetylation, myristoylation, ubiquitination, sumoylation and glycogen binding^20, 21^. It is unknown whether Epe1 is also involved in the regulation of these modification. Mass spectrometry analysis of AMPK subunits in *epe1*Δ and WT cells during stress responses is required to test this hypothesis.

## Methods

### Yeast strains and media

All the strains used in this study are listed in Supplementary Table S1. Gene deletion and tagging were performed by homologous recombination using a plasmid-based method^22^. Regular yeast extract medium with supplements (YES) containing 3% glucose and SPAS medium was prepared as describe before^23^.

### Fivefold serial dilution assay and growth curve analysis

Overnight culture was diluted into fresh YES to an OD_600_ of 0.2. Cells were grown at 32 °C and were collected till OD_600_ reached 0.8. For serial dilution assay, cells were collected and adjusted to an OD_600_ of 1.0. Samples were diluted by fivefold for five times. 5 μl dilutions were spotted onto YES medium containing indicated concentrations of glucose, 0.1 M NaCl, or at pH 10.5. Plates were incubated for 2 or 3d at 32 °C before imaging. For growth curve analysis, cells were washed and resuspended in YES liquid medium containing indicated concentrations of glucose, 0.05 M NaCl, or at pH 10.5, to start at an OD_600_ of 0.08. Cells were grown at 32 °C and OD_600_ of the culture was measured at indicated time points. Experiments were performed in triplicates.

### RT-PCR

Overnight culture was diluted into fresh YES to an OD_600_ of 0.2. 1 × 10^8^ cells were collected after growing for 10 h. Total RNA were extracted by using the RiboPure Yeast (AM1926, Life Technologies, Carlsbad, CA, USA) and reverse transcribed into cDNA by using PrimeScript RT (RR037A, Takara, Dalian, China). qPCR was performed using SYBR Premix Ex TaqII (RR820A, Takara) in a LightCycler 480 II Real-Time PCR System (Roche Applied Science, Penzberg, Upper Bavaria, Germany). Primers used are listed in Supplementary Table S2.

### Whole cell extract and fraction preparation

Cells were grown in YES overnight. Cells were harvested and washed by PBS for three times. Cells were then resuspended to an OD_600_ of 0.2 in fresh YES liquid medium containing indicated concentrations of glucose, 0.1 M NaCl, 0.03% H_2_O_2_, or at pH 11. Cells were grown at 32 °C and were collected till OD_600_ reached 0.6~0.8. To induce depletion of nutrients, cells were collected after growing in standard YES liquid medium for 4, 10 and 15 h. 5 × 10^8^ cells were pelleted and washed with lysis buffer (1 M Tris-HCl (pH 8.0), 167 mM NaCl, 1.2 mM EDTA, 1% TritonX-100, 0.1% Na-deoxycholate, 20 mM NaF, 2 mM phosphoglycerol). Cells were resuspended in lysis buffer containing protease inhibitors cocktail (05892970001, Roche Applied Science, Penzberg, Upper Bavaria, Germany) and homogenized with a bead-beater (FastPrep-24, MP, California, USA) by glass beads. Lysate was centrifuged. Supernatant was supplemented with 4xSDS-PAGE loading buffer and then boiled. Cytoplasmic and nuclear fractions were prepared from 5 × 10^8^ cells by using Minute Cytoplasmic & Nuclear Extraction Kits (SC-003, Invent Biotechnologies, Inc, Plymouth, MN, USA). Elution from different fractions was boiled with 4xSDS-PAGE loading buffer. Samples were subjected to Western blot by using following antibodies: anti-HA (M20003L, Abmart, Shanghai, China), anti-Phospho-AMPKα (Thr172) (2531, Cell Signaling Technology, Boston, MA, USA), anti-Tubulin (T8203, Sigma-Aldrich, St Louis, MO, USA). The blots were scanned by GeneGnome HR system (Syngene, Cambridge, UK).

### Co-immunoprecipitation

Overnight culture was dilute into fresh YES to an OD_600_ of 0.2. 5 × 10^10^ cells were harvested after growing for 10 h. Cells were washed and then resuspended in Buffer 1 supplemented with protease inhibitors as described above. Cells were broken by a high-pressure cell homogenizer (JN-02C, JNBIO, Guangzhou, China) and supernatant were collected after centrifugation. Supernatant was incubated with anti-HA antibody and then protein A/G PLUS-agarose beads (sc-2003, Santa Cruz, Dallas, TX, USA) for 6 h. Bead-conjugated complexes were washed with lysis buffer, boiled in 1xSDS-PAGE loading buffer and subjected to Western blot.

### Mating efficiency and iodine staining

WT *h* ^*90*^ and mutant strains were growing in YES overnight. Cells were harvested and washed by PBS for three times. Cells were resuspended in H_2_O to an OD_600_ of 60. 10 μl suspension was spotted onto SPAS plate and grown at 32 °C for 24 h. Cells were observed by a DIC microscope (IX51, Olympus, Tokyo, Japan). Percentages of zygotes were calculated as described before^24^. The spots on the medium were stained by iodine vapor for 2.5 min and then imaged.

### Preparation and quantification of fatty acids

Overnight culture was dilute into fresh YES to an OD_600_ of 0.2. 5 × 10^8^ cells were collected after growing for 10 h. Total fatty acid was prepared and quantified by gas chromatography–mass spectrometry as describe before^25^. Whole cell extract was prepared as described above. Amount of acetyl-CoA in the extract was quantified by mouse acetyl-CoA ELISA Kit (ABIN457115, www.antibodies-online.cn), and that of malonyl-CoA by mouse malonyl coenzyme A ELISA Kit (ABIN366452, www.antibodies-online.cn). Statistical significance was analyzed by student’s *t*-test using Excel.

## Electronic supplementary material

Supplementary information
