# Supplementary material for: Epe1 contributes to activation of AMPK by promoting phosphorylation of AMPK alpha subunit, Ssp2
Source: Sci Rep. 2017 Jun 9;7:3208. doi: 10.1038/s41598-017-03442-0 (PMC5466600; doi:10.1038/s41598-017-03442-0)
Supplement: Supplementary file 1 — Supplementary information [file 41598_2017_3442_MOESM1_ESM.doc]

**Supplementary Information**

**Epe1 contributes to activation of AMPK by promoting phosphorylation of AMPK alpha subunit, Ssp2**

Yongyi Chen^1,2^, Xiaoyue Hu^1,2^, Chao Guo^1,2^, Yao Yu^1,2,*^, Lu Hong^1,2,3,*^

^1^ State Key Laboratory of Genetic Engineering, School of Life Sciences, Fudan University

^2^ Shanghai Engineering Research Center of Industrial Microorganisms, Shanghai, China, 200438

^3^ Shanghai Collaborative Innovation Center for Biomanufacturing Technology, Shanghai, China, 200237

*Correspondence and requests for materials should be addressed to Y.Y. (email: [yaoyu@fudan.edu.cn](mailto:yaoyu@fudan.edu.cn)) or H.L. (email: honglu0211@yahoo.com)

**
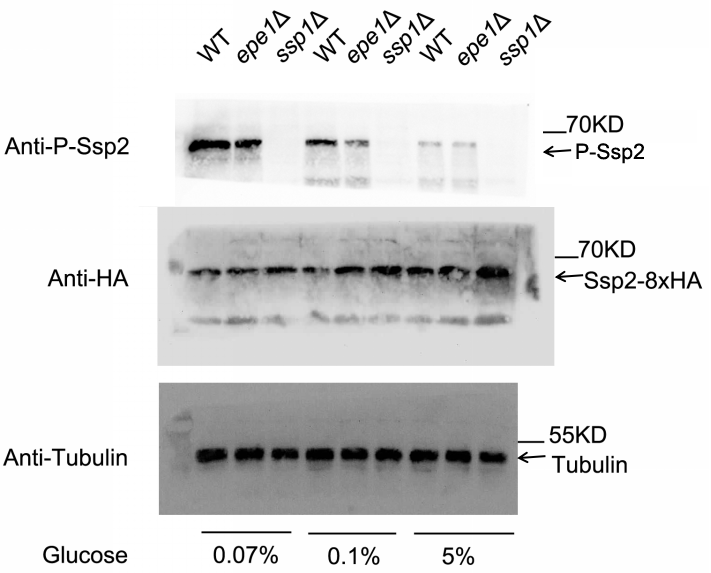
**

**Supplementary Figure S1. Full-size blots of Fig. 2a.**

Western blot to examine the amount of phosphorylated Ssp2 (P-Ssp2) and Ssp2-8HA in WT, *epe1*Δ and *ssp1*Δ cells growing in YES containing different concentrations of glucose. Tubulin was detected as a loading control.


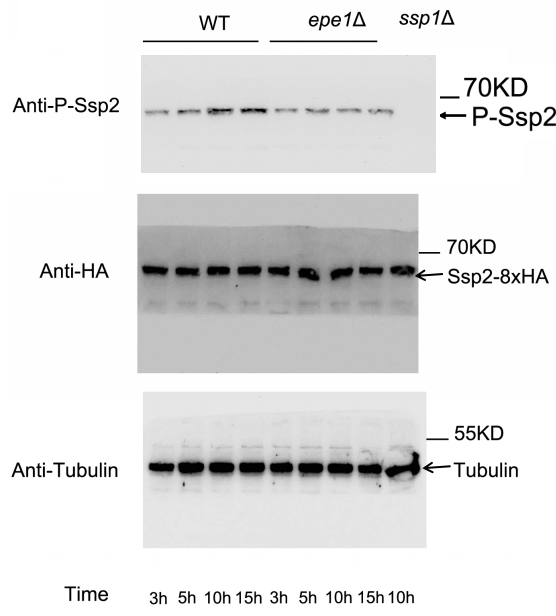


**Supplementary Figure S2. Full-size blots of Fig. 2b.**

Western blot to examine amount of phosphorylated Ssp2 (P-Ssp2) and Ssp2-8xHA in WT, *epe1*Δ and *ssp1*Δ cells after growing in YES for 3,5, 10 or 15h. Tubulin was detected as a loading control.


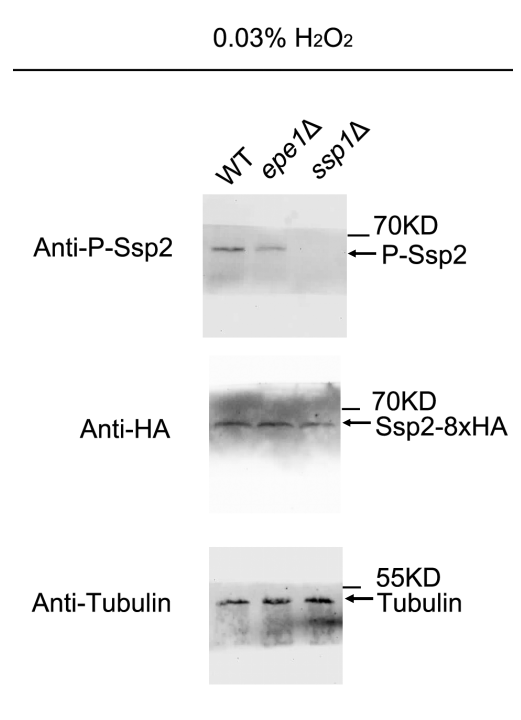


**Supplementary Figure S3. Full-size blots of Fig. 2c.**

Western blot to examine amount of phosphorylated Ssp2 (P-Ssp2) and Ssp2-8xHA in WT, *epe1*Δ and *ssp1*Δ cells growing in YES containing 0.03% H_2_O_2_. Tubulin was detected as a loading control.


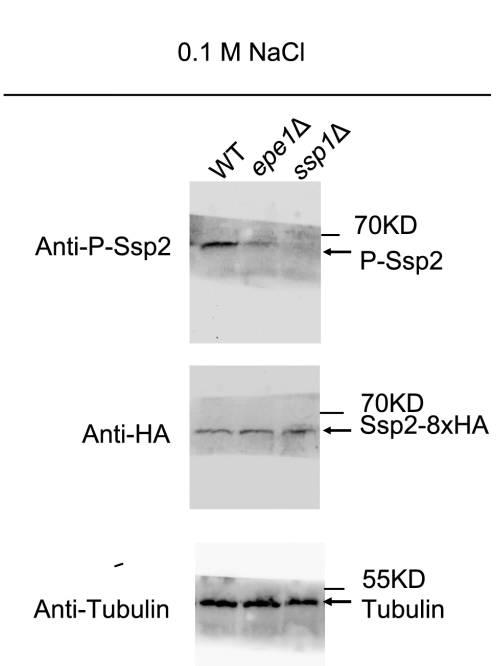


**Supplementary Figure S3. Full-size blots of Fig. 2d.**

Western blot to examine amount of phosphorylated Ssp2 (P-Ssp2) and Ssp2-8xHA in WT, *epe1*Δ and *ssp1*Δ cells growing in YES containing 0.1 M NaCl. Tubulin was detected as a loading control.


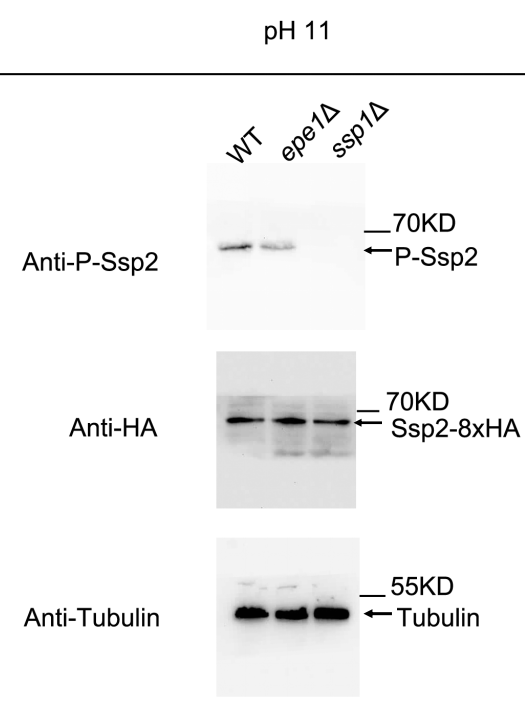


**Supplementary Figure S5. Full-size blots of Fig. 2e**

Western blot to examine amount of phosphorylated Ssp2 (P-Ssp2) and Ssp2-8xHA in WT, *epe1*Δ and *ssp1*Δ cells growing in YES at pH 11. Tubulin was detected as a loading control.


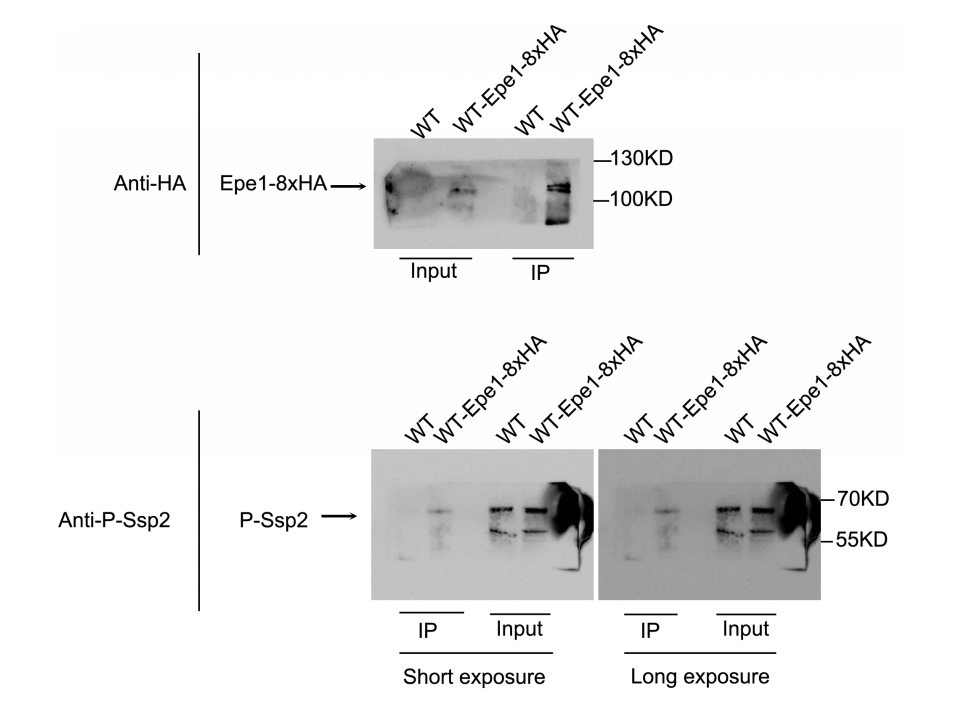


**Supplementary Figure S6. Full-size blots of Fig. 3b.** Co-immunoprecipitation assay to analyze the association between Epe1 and Ssp2. Epe1-8xHA immunoprecipitation was followed by the Western blot of phosphorylated Ssp2 (P-Ssp2). Input of P-Ssp2 was cropped from a blot with short exposure, while IP of P-Ssp2 was cropped from the same blot with long exposure.


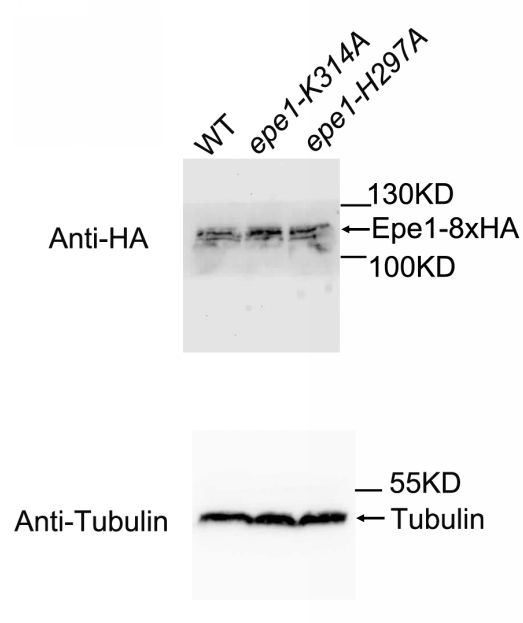


**Supplementary Figure S7. Full-size blots of Fig. 3d**

Western blot to examine the protein levels of Epe1, Epe1-K314A and Epe1-H297A. Tubulin was detected as a loading control.


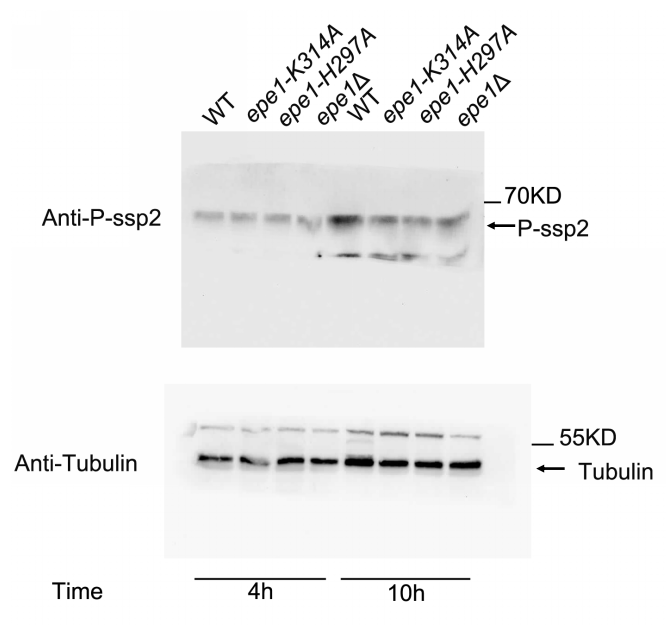


**Supplementary Figure S8. Full-size blots of Fig. 3e.**

Western blot to examine amount of P-Ssp2 in WT, *epe1*Δ, *epe1-K314A* and *epe1-H297A* cells after growing in YES for 4 or 10h. Tubulin was detected as a loading control.


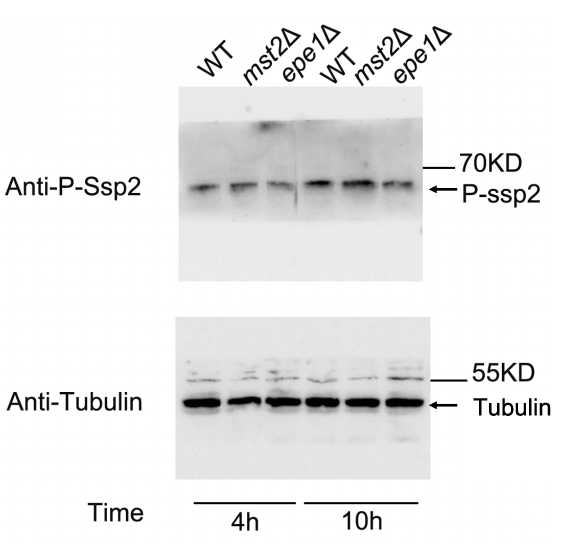


**Supplementary Figure S9. Full-size blots of Fig. 3f.**

Western blot to examine amount of P-Ssp2 in WT, *mst2*Δ and *epe1*Δ cells after growing in YES for 4 or 10h. Tubulin was detected as a loading control.


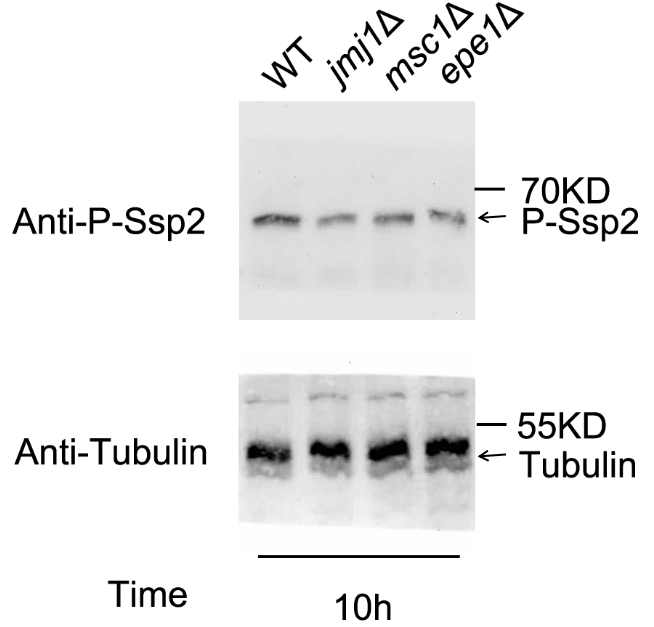


**Supplementary Figure S10. Full-size blots of Fig. 4b.**

Western blot to examine amount of P-Ssp2 in WT, *jmj1*Δ,*msc1*Δ and *epe1*Δ cells after growing in YES for 10h. Tubulin was detected as a loading control.


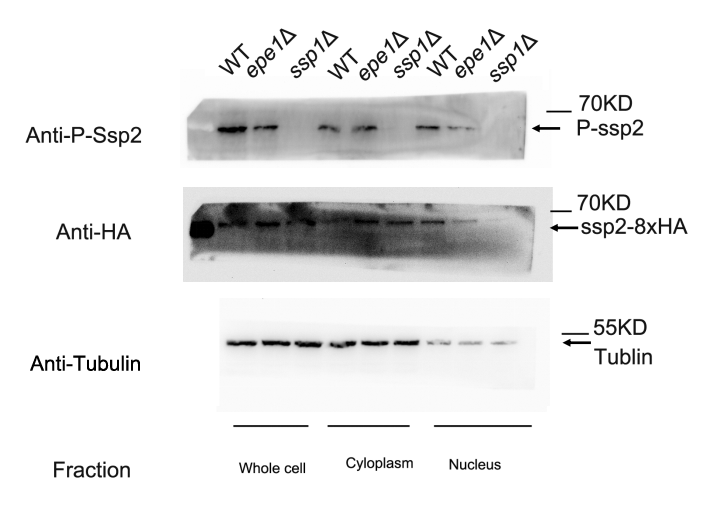


**Supplementary Figure S11. Full-size blots of Fig. 5a.**

Western blot to examine the amount of phosphorylated Ssp2 (P-Ssp2) and Ssp2-8XHA in the cytoplasmic and nuclear fractions from WT, *epe1*Δ, *ssp1*Δ or *ssp2*Δ cells after growing in YES for 10h. Tubulin was detected as a loading control.

**Supplem**entary Table S1. List of strains used in this study.

| **Strain** | **Genotype** | **Source** |
| --- | --- | --- |
| LHP164 | *h^+^,leu1-32,ade6-m210,ura4-D18* | Bioneer^1^ |
| LHP477 | *h^+^,leu1-32,ade6-m210,ura4-D18, epe1*Δ*::kanMX6* | Bioneer |
| LHP478 | *h^+^,leu1-32,ade6-m210,ura4-D18,mst2*Δ*::kanMX6* | Bioneer |
| LHP479 | *h^+^,leu1-32,ade6-m210,ura4-D18, ssp2*Δ::*hphMX4* | This study |
| LHP480 | *h^+^,leu1-32,ade6-m210,ura4-D18, ssp1*Δ::*hphMX4* | This study |
| LHP481 | *h^+^,leu1-32,ade6-m210,ura4-D18, epe1*Δ::*natMX4* | This study |
| LHP482 | *h^+^,leu1-32,ade6-m210,ura4-D18, epe1*Δ::*natMX4, ssp2-8HA*::*kanMX6* | This study |
| LHP483 | *h^+^,leu1-32,ade6-m210,ura4-D18, ssp1*Δ::*hphMX4, ssp2-8HA*::*kanMX6* | This study |
| LHP484 | *h^+^,leu1-32,ade6-m210,ura4-D18, epe1-8HA*::*kanMX6* | This study |
| LHP485 | *h^+^,leu1-32,ade6-m210,ura4-D18, epe1-K314A-8HA*::*hphMX4* | This study |
| LHP486 | *h^+^,leu1-32,ade6-m210,ura4-D18, epe1-H297A-8HA*::*hphMX4* | This study |
| FY7266 | *h^90^, ade6-M210, ura4-D18 leu1-32* | YGRC^2^ |
| LHP487 | *h^90^, ade6-M210, ura4-D18 leu1-32, epe1*Δ::*natMX4* | This study |
| LHP488 | *h^90^, ade6-M210, ura4-D18 leu1-32, ssp1*Δ::*hphMX4* | This study |
| LHP489 | *h^90^, ade6-M210, ura4-D18 leu1-32, ssp2*Δ::*hphMX4* | This study |
| LHP490 | *h^+^,leu1-32,ade6-m210,ura4-D18, jmj1*Δ*::kanMX6* | Bioneer |
| LHP491 | *h^+^,leu1-32,ade6-m210,ura4-D18, jmj2*Δ*::kanMX6* | Bioneer |
| LHP492 | *h^+^,leu1-32,ade6-m210,ura4-D18, jmj4*Δ*::kanMX6* | Bioneer |
| LHP493 | *h^+^,leu1-32,ade6-m210,ura4-D18, msc1*Δ*::kanMX6* | Bioneer |
| LHP494 | *h^+^,leu1-32,ade6-m210,ura4-D18, jmj1*Δ*::kanMX6, epe1*Δ::*natMX4* | This study |
| LHP495 | *h^+^,leu1-32,ade6-m210,ura4-D18, jmj2*Δ*::kanMX6, epe1*Δ::*natMX4* | This study |
| LHP496 | *h^+^,leu1-32,ade6-m210,ura4-D18, jmj4*Δ*::kanMX6, epe1*Δ::*natMX4* | This study |
| LHP497 | *h^+^,leu1-32,ade6-m210,ura4-D18,msc1*Δ*::kanMX6, epe1*Δ::*natMX4* | This study |
| LHP498 | *h^+^,leu1-32,ade6-m210,ura4-D18, ssp2-8HA*::*kanMX6* | This study |

^1^ Strain was purchased from Bioneer (https://us.bioneer.com). Identity of mutant was verified by PCR.

^2^ Strain was purchased from Yeast Genetic Resource Center (YGRC) (http://yeast.lab.nig.ac.jp/yeast/).

**Supplementary Table S2. Primers used in this study**

| **Primer name** | **Sequence** | **Used for** |
| --- | --- | --- |
| *ssp2*^+^ forward | AGGCTCTTATGCTGATTCACG | RT-PCR |
| *ssp2*^+^ reverse | ACAATGTAATCCTCCGAGAACC | RT-PCR |
| *ssp1*^+^ forward | GATTAGCGAGGCAAGACAATAC | RT-PCR |
| *ssp1*^+^ reverse | CACAATTAGAAGAGTTAAGGAGAAGG | RT-PCR |
| *amk2*^+^ forward | CACAACAACGCCTGACAATG | RT-PCR |
| *amk2*^+^ reverse | CCGCCTTCCTGCTGATTATC | RT-PCR |
| *cbs2*^+^ forward | GTCTATGAATCGGTGGATGTAATG | RT-PCR |
| *cbs2*^+^ reverse | TCAATAAAGCCTCGCCTACG | RT-PCR |
| *fbp1*^+^ forward | GGCAGTCAAGGCGATATTAGC | RT-PCR |
| *fbp1*^+^ reverse | CAGCAGCAACCATTTCTTTACC | RT-PCR |
